# miR-218-5p and doxorubicin combination enhances anticancer activity in breast cancer cells through Parkin-dependent mitophagy inhibition

**DOI:** 10.1038/s41420-024-01914-7

**Published:** 2024-03-21

**Authors:** Francesco Davide Naso, Krenare Bruqi, Valeria Manzini, Valerio Chiurchiù, Mara D’Onofrio, Ivan Arisi, Flavie Strappazzon

**Affiliations:** 1 IRCCS Santa Lucia Foundation, Via del Fosso di Fiorano 64/65, 00143 Rome, Italy; 2 European Brain Research Institute (EBRI) “Rita Levi-Montalcini”, Viale Regina Elena 295, 00161 Rome, Italy; 3 Institute of Translational Pharmacology, CNR, Via del Fosso del Cavaliere, 100, 00133 Rome, Italy; 4 Laboratory of Resolution of Neuroinflammation, IRCCS Santa Lucia, Foundation, Via del Fosso di Fiorano 64/65, 00143 Rome, Italy; 5 Physiopathologie et Génétique du Neurone et du Muscle, UMR5261, U1315, Institut NeuroMyogène, Univ Lyon, Univ Lyon 1, CNRS, INSERM, 69008 Lyon, France; 6 Present Address: Physiopathologie et Génétique du Neurone et du Muscle, UMR5261, U1315, Institut NeuroMyogène, Univ Lyon, Univ Lyon 1, CNRS, INSERM, 69008 Lyon, France; 7 Present Address: Department of Biology and Biotechnologies “Charles Darwin”, Sapienza University of Rome, Piazzale Aldo Moro 5, 00185 Rome, Italy

**Keywords:** Mitophagy, Breast cancer

## Abstract

Breast Cancer (BC) is one of the most common tumours, and is known for its ability to develop resistance to chemotherapeutic treatments. Autophagy has been linked to chemotherapeutic response in several types of cancer, highlighting its contribution to this process. However, the role of mitophagy, a selective form of autophagy responsible for damaged mitochondria degradation, in the response to therapies in BC is still unclear. In order to address this point, we analysed the role of mitophagy in the treatment of the most common anticancer drug, doxorubicin (DXR), in different models of BC, such as a luminal A subtype-BC cell line MCF7 cells, cultured in 2-Dimension (2D) or in 3-Dimension (3D), and the triple negative BC (TNBC) cell line MDA-MB-231. Through a microarray analysis, we identified a relationship between mitophagy gene expressions related to the canonical PINK1/Parkin-mediated pathway and DXR treatment in BC cells. Afterwards, we demonstrated that the PINK1/Parkin-dependent mitophagy is indeed induced following DXR treatment and that exogenous expression of a small non-coding RNA, the miRNA-218-5p, known to target mRNA of Parkin, was sufficient to inhibit the DXR-mediated mitophagy in MCF7 and in MDA-MB-231 cells, thereby increasing their sensitivity to DXR. Considering the current challenges involved in BC refractory to treatment, our work could provide a promising approach to prevent tumour resistance and recurrence, potentially leading to the development of an innovative approach to combine mitophagy inhibition and chemotherapy.

## Introduction

Doxorubicin (DXR, also called Adriamycin [1]), is one of the most popular chemotherapeutic drugs currently used to treat several types of solid and haematological tumours. This antibiotic of the anthracycline group has the ability to inhibit Topoisomerase II and cause DNA damage, interfering with its metabolism [2]. DXR is frequently used in breast cancer (BC) treatment, alone or in combination with other chemotherapeutic drugs. However, the development of resistance, despite its high toxicity, reduces the efficacy of the therapy. In addition to its nuclear targets, DXR affects other cellular structures such as mitochondria. Indeed, it has been shown that DXR selectively binds to cardiolipin, an inner mitochondrial membrane (IMM) phospholipid, interfering with the electron transport chain and inducing reactive oxygen species (ROS) generation and mitochondrial damage [3, 4]. The main cellular response to mitochondrial damage is the mitophagy process, a selective autophagic degradation of mitochondria that ensures a quality control of the organelles [5]. In mammalian cells, the common mitophagy pathway activated after mitochondrial damage is governed by the PTEN induced kinase 1(PINK1)/Parkin axis [6]. PINK1 is a serine/threonine kinase that is stabilised on the surface of damaged mitochondria, where it phosphorylates several outer mitochondrial membrane (OMM) proteins. The E3 ubiquitin (ub) ligase Parkin exists in an auto-inhibited form in the cytosol and PINK1 activates it in two different ways: 1) through direct phosphorylation on its ub-like-domain, enhancing Parkin activity; 2) through phosphorylation of the ub chains on OMM proteins, favouring Parkin translocation on mitochondria [7]. After activation, Parkin adds ub-chains on OMM proteins allowing the detection of damaged mitochondria by several mitophagy receptors [8] that bring the organelles into a double-membrane vesicle, the so called autophagosome which next fuse with the lysosome for degradation of its content [9]. Mitophagy has been detected in colon cancer cells after DXR administration [10] and in lung cancer cells in response to cisplatin, etoposide or UV irradiation [11]. In addition, this process has been recently characterised in osteosarcoma and ovarian cancer as a mediator of resistance to cisplatin [12]. All these works highlight the fact that mitophagy could be a canonical cellular response to cancer therapies. In the context of BC, some studies have shown that inhibition of autophagy improved the sensitivity to DXR [13, 14]. In fact, an increased sensitivity to DXR of BC cells accompanied with auto/mitophagosomes accumulation has been shown, suggesting that block of autophagy/mitophagy may work in synergy to this drug [15]. However, information about a potential mitophagy induction in BC cells in response to DXR treatment is currently missing.

In this study we discovered that the PINK1/Parkin-mediated mitophagy pathway is stimulated following DXR treatment in MCF7 BC cells cultured in 2-Dimension (2D) or in 3-Dimension (3D), and in MDA-MB-231 cells, suggesting that mitophagy genes involved in this process could be novel targets for BC treatment. Since evidence suggests microRNA-218-5p (miR-218-5p) as an onco-suppressor in several cancers [16–18], by targeting Parkin mRNA [19], we thought to use this tool to inhibit DXR-induced mitophagy. We indeed demonstrated that, blocking Parkin through miR-218-5p expression, increases cell sensitivity to DXR treatment in luminal A MCF7 and in Triple-Negative BC (TNBC) MDA-MB-231 cells.

Altogether, our work increases knowledge on the role played by mitophagy in response to chemotherapy and demonstrates the efficacy of a specific Parkin-mediated mitophagy inhibition in improving sensitivity to DXR treatment in BC cells. Considering the current challenges involved in BC refractory treatment, this work could provide a promising approach to modifying the tumour response to the drug and to prevent resistance and relapses.

## Results

### Relationship between mitophagic genes expression and DXR treatment in MCF7 BC cells

**Fig. 1:**
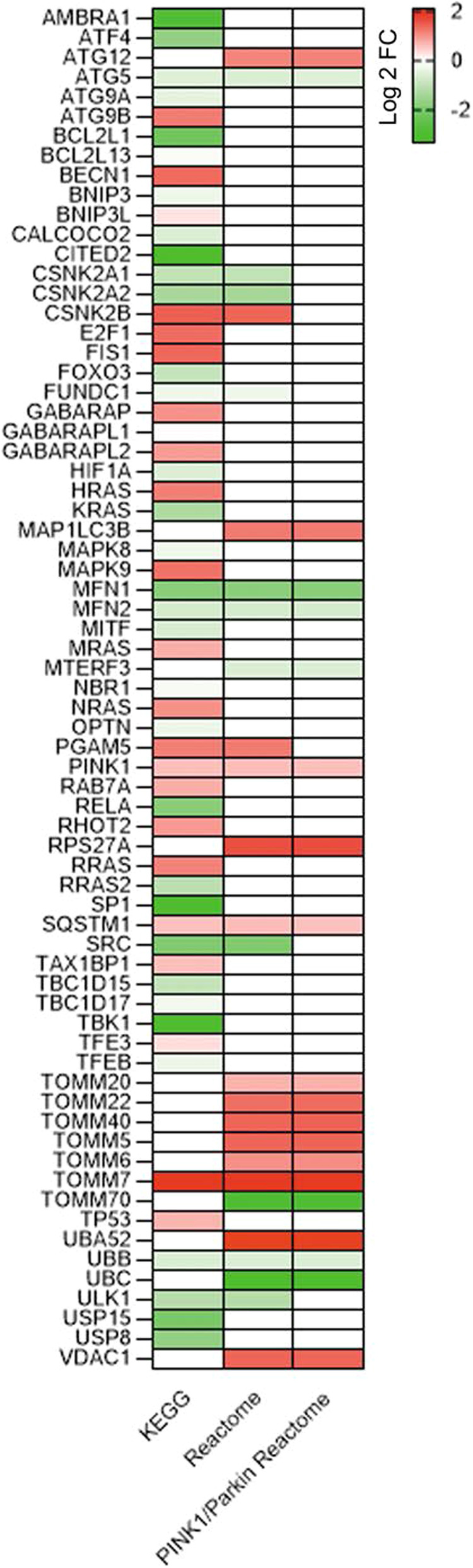
DXR treatment induces modulation of mitophagy-associated genes expression. Heatmap show down (green) and up (red) regulated genes, expressed as fold change (FDR < 0.05; Log2 fold-change ratio >1.0), after DXR treatment. Gene lists “Mitophagy” were from KEGG (first column) and from Reactome (second column). Gene list “PINK1/Parkin-mediated mitophagy” in the third column was from Reactome. Colour intensity is proportional to the individual level of expression (see reference on the right). White squares are genes not listed in the referred gene list.

In order to investigate whether mitophagic genes could be modulated in BC cells in response to DXR treatment, we first performed a microarray analysis on total RNA extracted from cells treated with DXR for 24 h. We chose the luminal-A sub-type BC cell line MCF7 as model system for its sensitivity to DXR, the better condition to evaluate the modulation of functions after drug administration. We scored gene lists from KEGG and Reactome databases, with a focus on “mitophagy-related genes”. Interestingly, genes involved in autophagy such as ATG12, ATG9, BECN1, MAP1LC3B appeared up-regulated in DXR treatment condition. In addition, genes involved in mitophagy, like BNIP3L (also called NIX) and PINK1 were up-regulated following DXR treatment suggesting a putative role of mitophagy in response to DXR (Fig. 1). To reinforce these findings, we performed a bioinformatical analysis by extracting specific gene list from public datasets of gene expression microarray experiments performed in MCF7 cells treated with DXR, or resistant to DXR, compared to untreated samples. In accordance with our results, we found that some auto/mitophagic genes, were modulated in response to DXR treatment (Supplementary Fig. 1). These data confirm that autophagy is most likely activated in response to DXR in BC cells [20], and that some mitophagic proteins are up-regulated after DXR treatment.

### DXR induces PINK1/Parkin-dependent mitophagy in MCF7 cell line

**Fig. 2:**
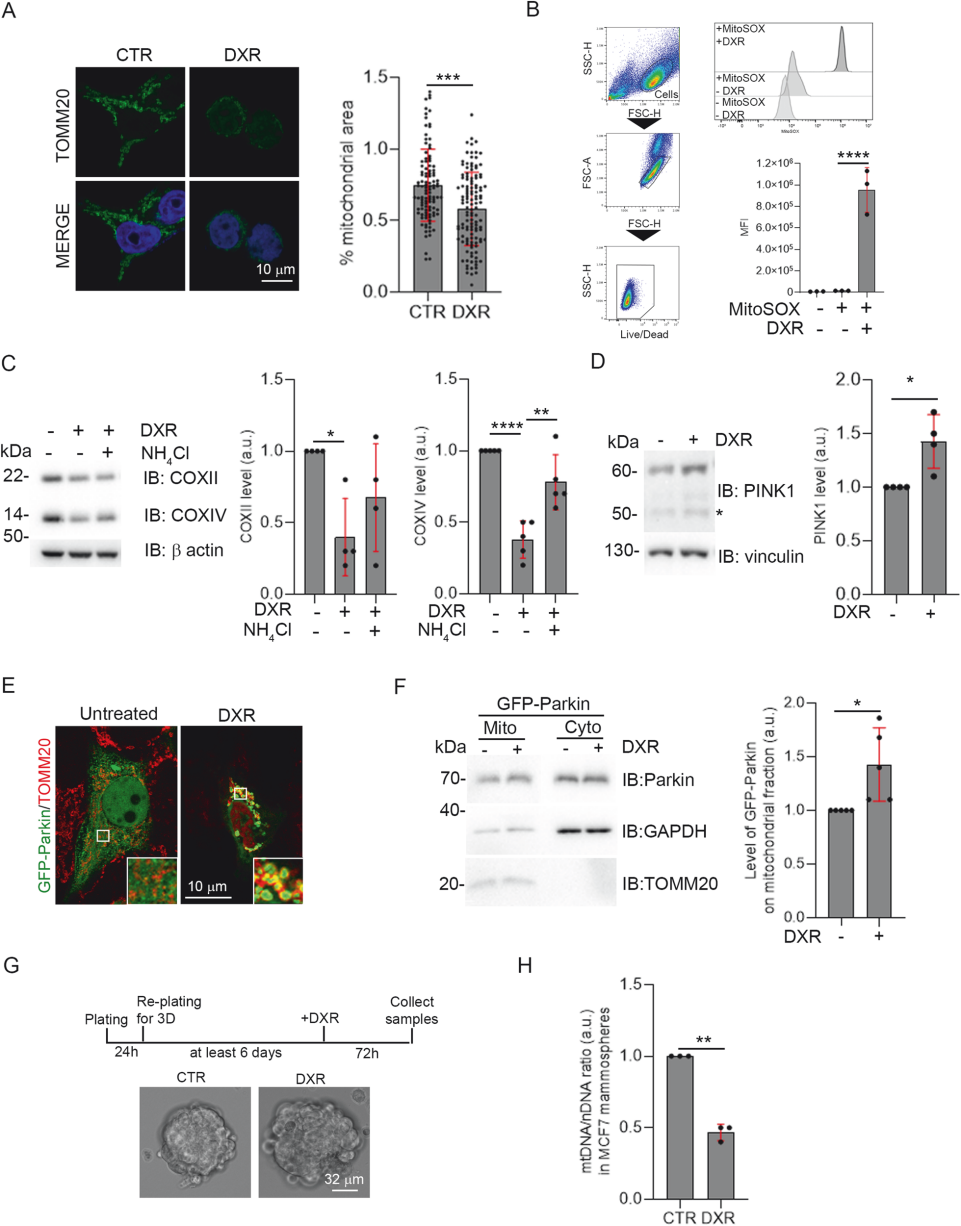
DXR treatment induces PINK1/Parkin-mediated mitophagy. **A** Representative images of the mitochondrial network of MCF7 cells visualised through the TOMM20 staining, in the indicated conditions. Each dot in the graph represents the cellular area occupied by mitochondria expressed as a percentage (%) for each cell analysed, after normalisation with the mean area +1S.D. in CTR condition, of each experiment. Scale bar, 10 μm is shown. At least 50 cells from 3 independent experiments were analysed. **B** Flow cytometry plots and histograms show the mean fluorescent intensity (MFI) of MitoSOX in the indicated conditions. **C** Total lysate from MCF7 treated with DXR 30 µM for 24 h with and without NH_4_Cl was immunoblotted for the indicated proteins. The graphs show the mitochondrial markers COXII and COXIV protein levels normalised on β-actin that has been used as a loading control. Solid dots represent the value respect the control condition, from 4 and 5 (for COXII and COXIV respectively) independent experiments. Pictures show the representative image of the signals. **D** Histograms show the mean of the signal of PINK1 protein, normalised on Vinculin that has been used as a loading control, in MCF7 after 1 h of DXR 30 µM treatment. Solid dots represent the value respect to the control condition, from 4 independent experiments. The “*” symbol in the image indicates the signal of the cleaved form of PINK1, reported as cytosolic and that was not considered for the quantification. **E** Representative images of GFP-Parkin translocation on mitochondria are illustrated on the right panels. Scale bar, 10 μm is shown. **F** The graph indicates the mean of the signal of GFP-Parkin protein in mitochondrial fractions, after 2 h of DXR 30 µM treatment. Solid dots represent the value respect to the control condition, from 5 independent experiments. Pictures show the representative image of the signals. TOMM20 and GAPDH have been used, respectively, as a loading control for mitochondrial and cytosolic fractions. **G** Protocol used in order to obtain MCF7 grown as mammospheres, depicted in the image in treated and untreated conditions. Scale bar, 32 μm is shown. **H** Measure of mtDNA/nDNA *ratio* in 3D MCF7 cultures after 72 h of DXR 2 µM treatment. Dots represent the mean of Ct of at least 3 technical replicates, from 3 independent experiments. S.D. are indicated in red. Mann-Whitney test was performed for **A**; unpaired t-test (Welch’s correction) was performed for **D**, **E** and **G**. One-way ANOVA (Tukey’s multiple comparison) test was performed for **B** and **C**. *p < 0.05, **p < 0.01, ***p < 0.001, ****p < 0.0001.

To test the hypothesis that DXR could stimulate mitophagy in BC cells, we performed a confocal microscopy analysis on MCF7 cells treated or not with DXR to visualise the mitochondrial network by staining TOMM20. As illustrated in Fig. 2A, treatment of BC cells with DXR induces a reduction of the mitochondrial area within cells. Since damaged mitochondria are known to produce ROS, we measured their production in MCF7 through flow cytometry by evaluating the MitoSOX dye signal. High levels of mitochondrial ROS were assessed following DXR treatment, indicating compromised mitochondrial functions (Fig. 2B). Since mitophagy induction and/or inhibition of mitochondrial biogenesis are both characterised by a decreased of mitochondrial mass, we examined mitochondrial content in MCF7 cells treated with or without DXR and NH_4_Cl, an inhibitor of the autophagic clearance [21]. In particular, we monitored the levels of two IMM markers, the cytochrome c oxidase subunit II (COXII) and cytochrome c oxidase subunit IV (COXIV). NH_4_Cl co-treatment confirmed the effective arrest of the autophagic flux as illustrated by the accumulation of LC3-II dots (Supplementary Fig. 2A). Also, we observed decreases of both COXII and COXIV protein levels in MCF7 cells treated with DXR, a decrease of COXIV that is rescued by inhibiting the autophagic clearance and that is partially rescued for COXII, supporting the contribution of autophagy in this process (Fig. 2C). Altogether, our data indicate that DXR treatment, in addition to its well-known effects, induces autophagic mitochondrial clearance in MCF7 cells.

The main pathway regulating autophagic degradation of mitochondria in mammalian cells is governed by PINK1/Parkin axis [6]. In order to investigate its involvement in response to DXR, we checked for PINK1 protein stabilisation and for Parkin translocation on mitochondria, which are two indicators of this mitophagic pathway [7]. PINK1, whose expression is up-regulated in array and in silico analysis (Fig. 1; Supplementary Fig. 1), saw its protein level increased after 1 h of DXR treatment, confirming the stabilisation of the protein (Fig. 2D). We next sought to address the translocation of Parkin on mitochondria. Considering that Parkin is known to be weakly expressed in cancer cells [11] we first decided to evaluate the level of expression of this E3 Ub ligase in our BC cell lines. As illustrated in Supplementary Fig. 2B, we were able to detect endogenous Parkin in MCF7 and MDA-MB-231 cells. Moreover, despite the low levels of Parkin expression, we found an up-regulation of PRKN gene expression in MCF7 after treatment with DXR, in the unfiltered data of our microarray analysis (Supplementary Fig. 2C, D), reinforcing the hypothesis that the PINK1/Parkin pathway might be stimulated after DXR treatment. In order to easily monitor the translocation of Parkin in treated MCF7, we transfected the cells with a vector encoding GFP-tagged Parkin and observed an increase of Parkin protein on mitochondrial-enriched fractions after 2 h of DXR treatment (Fig. 2E, F). Finally, to verify a putative mitophagy induction following DXR treatment in a model of cancer stem-cell, we assessed mitochondrial DNA (mtDNA)/nuclear DNA (nDNA) ratio as a read-out of mitochondria degradation in 3D-growing MCF7 cells (Fig. 2G). This type of culture, in which the stem-like population is enriched [22], showed a reduction of mtDNA content in DXR condition, indicating that mitochondrial clearance is induced in 3D MCF7 cells following DXR treatment (Fig. 2H). Altogether, these data indicate that DXR induces a PINK1/Parkin-dependent mitophagy in the MCF7 cell line grown both in 2D and 3D cultures.

### Parkin emerges as a target of miR-218-5p to improve treatment of BC

**Fig. 3:**
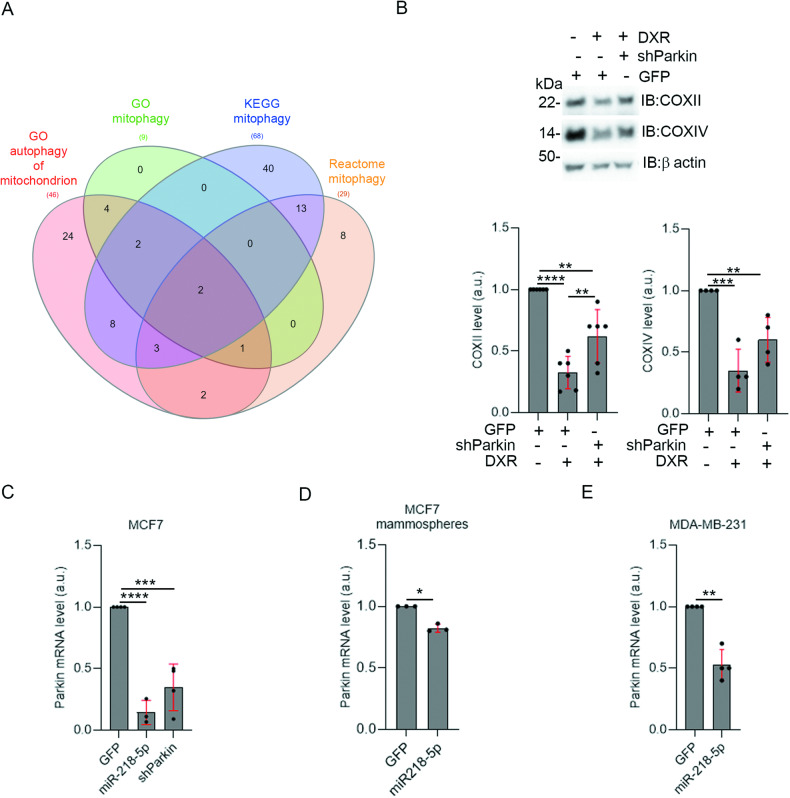
Parkin depletion inhibits DXR-induced mitophagy in MCF7. **A** A Venn diagram illustrates the intersection of four gene lists mitophagy-related (keywords “mitophagy” and “autophagy of mitochondrion”) from public databases AmiGO (Gene Ontology), KEGG and Reactome. The central intersection contains Parkin and SQSTM1 genes. **B** Total lysates from MCF7 cells transfected with GFP or GFP/shParkin vectors, and then treated with DXR 30 µM for 24 h, were immunoblotted with the indicated antibodies. The graphs show the mitochondrial markers COXII and COXIV protein levels normalised on β-actin that has been used as a loading control. Solid dots represent the value respect to the control condition, from from 6 and 4 (for COXII and COXIV respectively) independent experiments. **C**–**E** Level of Parkin mRNA in MCF7, in MCF7 grown as a mammospheres and in MDA-MB-231 after miR-218-5p expression, or shParkin expression in MCF7. Dots represent the mean of Ct of at least 3 technical replicates, from at least 3 (for **C**), 3(for **D**) and 4 (for **E**) independent experiments. S.D. are shown in red. One-way ANOVA (Tukey’s multiple comparison) test was performed for **B** and **C**. Unpaired t-test (Welch’s correction) was performed for **D** and **E** *p < 0.05, **p < 0.01, ***p < 0.001, ****p < 0.0001.

The general pro-survival role of autophagy in the cell combined with the fact that PINK1/Parkin-mediated mitophagy may play a role in the response to DXR treatment, support the hypothesis that targeting mitophagy may improve the effects of DXR. Interestingly, by combining four mitophagic gene lists from public databases (Gene Ontology, KEGG, Reactome), we found that Parkin and SQSTM1/p62 are the two common genes in all the analysed lists (Fig. 3A). This in silico analysis, together coupled with the increase in Parkin increased expression in unfiltered data from the microarray (Supplementary Fig. 2C, D) and the recruitment of GFP-Parkin on mitochondria after DXR administration (Fig. 2E, F), indicates that Parkin could be a putative gene to target in order to inhibit mitophagy activated by therapy. Indeed, by down-regulation of Parkin in MCF7 using specific shRNA, we were able to rescue the levels of both COXII and COXIV following DXR treatment, indicating that Parkin is a crucial actor in mediating the degradation of mitochondria following DXR treatment in BC cells (Fig. 3B). Since we previously demonstrated that miR-218-5p impairs Parkin-mediated mitophagy in HEK293 [19], and that its expression is inversely correlated with poor survival in BC patients [17], and since miRNA-based therapies are promising in cancer [23], we hypothesised that miR-218-5p expression could be useful to inhibit DXR-dependent mitophagy in BC cells, thus improving drug efficacy. To this aim, we reintroduced transiently miR-218-5p into MCF7 transfecting an inducible vector encoding the mature form of the miRNA, after administration of doxycycline (DOX). Indeed, miR-218-5p expression was efficient in decreasing Parkin mRNA level in MCF7 cells as much as using Parkin-specific shRNA (Fig. 3C). Interestingly, miR-218-5p expression was sufficient to reduce Parkin mRNA also in MCF7 mammospheres, although to a lesser extent than in 2D cultures probably due to the limited amount of miR-218-5p entering in 3D cultures, and in TNBC MDA-MB-231 (Fig. 3D, E). Consequently, restoring miR-218-5p expression inhibits Parkin levels in several sub-types of BC cells that correspond to more or less aggressive BC.

### miR-218-5p inhibits mitophagy induced by DXR treatment in BC cells

**Fig. 4:**
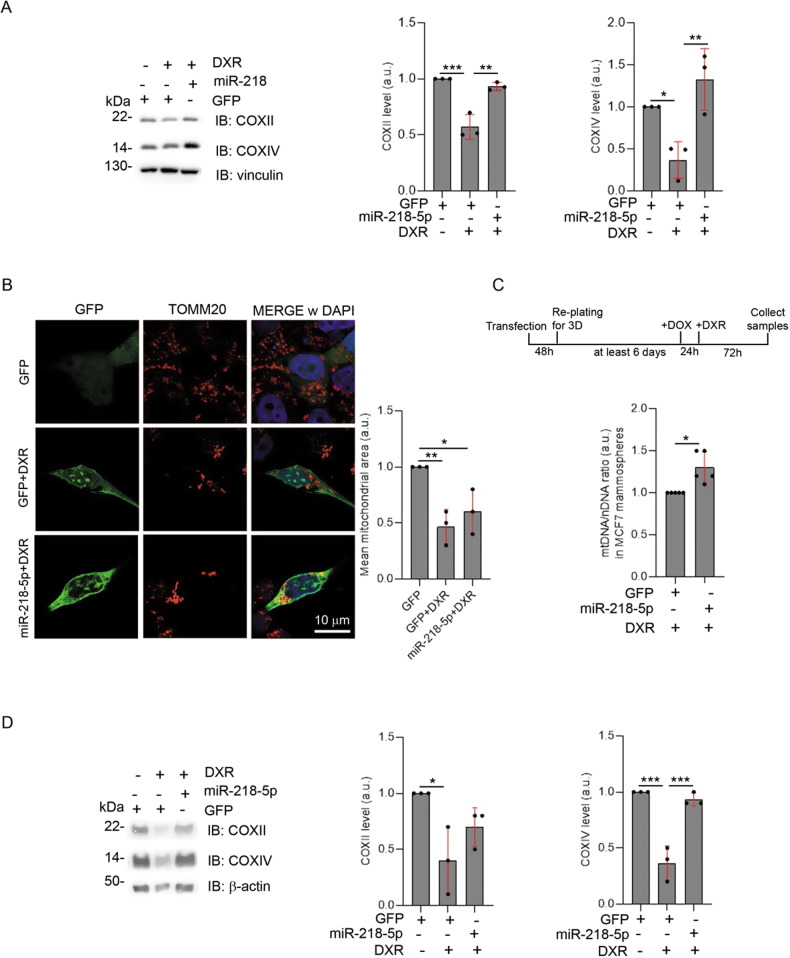
miR-218-5p impairs DXR-induced mitophagy. **A** Total lysate from MCF7 cells transfected with GFP or GFP/miR-218-5p vectors, and then treated with DXR 30 µM for 24 h, were immunoblotted with the indicated antibodies. The graphs show the mitochondrial markers COXII and COXIV protein levels normalised on Vinculin, which has been used as a loading control. Solid dots represent the value respect to the control condition, from 3 independent experiments. **B** Representative immunofluorescence images of the mitochondrial network, visualised through TOMM20 staining, in the indicated conditions. Histograms represent the mean of the cellular area occupied by mitochondria, expressed as %, for each cell analysed, respect to the control condition. At least, 25 cells from 3 independent experiments were analysed. Scale bar, 10 μm is shown. **C** mtDNA/nDNA *ratio* from MCF7 grown as mammospheres, expressing GFP or GFP/miR-218-5p, and treated with DXR 2 µM for 72 h. Dots represent the mean of Ct of at least 3 technical replicates, from 5 independent experiments. **D** Total lysate from MDA-MB-231 cells transfected with GFP or GFP/miR-218-5p and treated with DXR 30 µM for 24 h, were immunoblotted with the indicated antibodies. The graphs show the mitochondrial markers COXII and COXIV protein levels normalised on β-actin that has been used as a loading control. Solid dots represent the value respect the control condition, from 3 independent experiments. SD are shown on the graph in red. One-way ANOVA (Tukey’s multiple comparison) was performed for **A**, **B** and **D**. Unpaired t-test (Welch’s correction) was performed for **C**. *p < 0.05, **p < 0.01, ***p < 0.001.

At this point, we investigated whether miR-218-5p was capable of blocking mitophagy induced by DXR in BC. MCF7 cells overexpressing miR-218-5p, upon DXR treatment, appeared, indeed, unable to reduce their levels of COXII and COXIV mitochondrial markers, compared to control cells transfected with GFP alone (Fig. 4A). We strengthened our results by performing a confocal microscopy analysis of transfected GFP or GFP/miR-218-5p cells treated with DXR. Untreated cells showed a normal mitochondrial network (a typical appearance of the mitochondria observed in MCF7 cells) while upon DXR treatment, mitochondria network appeared fragmented and reduced. Again, expression of miR-218-5p, partially rescued mitochondrial mass, without reverting the fragmentation of the network (Fig. 4B), likely due to the down-regulation of mitofusin1 and 2 that we observed in the microarray analysis (Fig. 1). Being able to inhibit mitophagy-induced by DXR through the use of miR-218-5p expression in MCF7 cells, we wondered whether this miRNA was capable to limit DXR-induced mitochondria degradation also in the most aggressive conditions of the disease, such as in cancer-stem-like MCF7 cells and in MDA-MB-231. Indeed, these cells are known to be hard to target with chemotherapeutic treatments [24]. In order to investigate this, we transfected MCF7 with GFP or GFP/miR-218-5p encoded vectors and we next generated the mammospheres. Finally, 24 h before the DXR administration, we induced the expression of miRNA by treating cells with DOX (Fig. 4C). After treatment with DXR, miR-218-5p expression resulted in a small but significant retention of mtDNA after drug administration, indicating an altered mitochondrial clearance (Fig. 4C). Finally, we found decreases in both COXII and COXIV following DXR treatment also in MDA-MB-231 cells. Interestingly, the level of COXIV was rescued by expressing miR-218-5p and the levels of COXII partially rescued (Fig. 4D). Consequently, our results underline miR-218-5p as an inhibitor of DXR-mediated mitophagy both in stem and non-stem MCF7 BC cells and in TNBC cells.

### Mitophagy inhibition through miR-218-5p expression improves the sensitivity to DXR in MCF7 and MDA-MB-231 cells

**Fig. 5:**
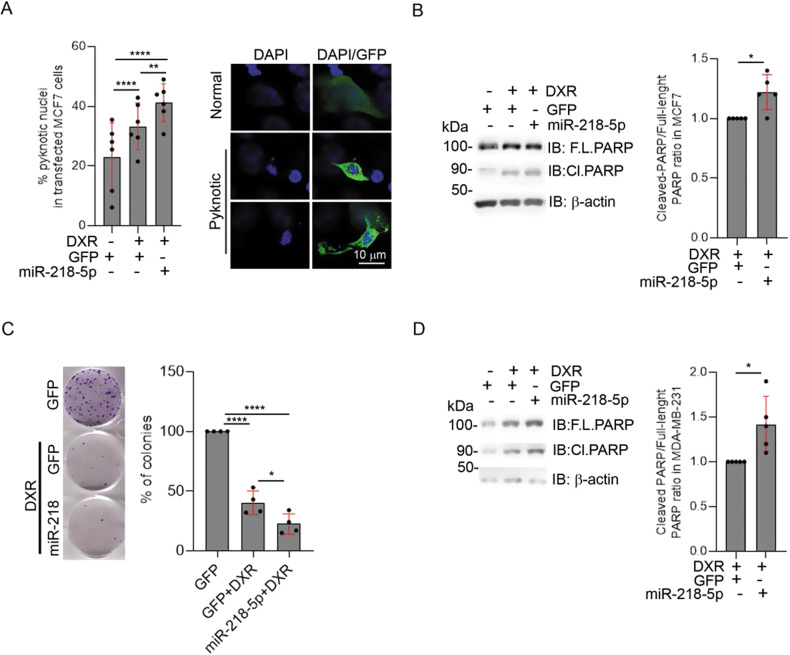
miR-218-5p expression enhances DXR anti-cancer effect. **A** The percentage (%) amount of pyknotic nuclei in transfected MCF7 cells (GFP positive), scored in the indicated condition, after treatment of DXR 10 µM for 48 h, have been reported in the graph. An example of the “pyknotic nuclei” considered in this analysis is illustrated on the right panel. Scale bar, 10 μm is shown. At least 482 nuclei, from 6 independent experiments, were analysed. **B** Histograms show the relative levels of cleaved-PARP in MCF7 expressing GFP or miR-218-5p after 12 h of DXR 1 µM treatment, normalised on total PARP. Solid dots represent the value of indicated conditions, from 5 independent experiments. F.L.PARP full length PARP, Cl.PARP cleaved PARP. **C** Colony formation assay highlights the ability of cells to form colonies in cells expressing GFP or GFP/miR-218-5p, treated with DXR 1 µM for 12 h. The graph represents the % of colonies obtained compared to the control condition. Solid dots represent the value of indicated conditions, from 4 independent experiments. **D** Histograms show the relative levels of cleaved-PARP in MDA-MB-231 cells expressing GFP or miR-218-5p, treated with DXR 1 µM for 12 h, normalised on total PARP. Solid dots represent the value of indicated conditions, from 5 independent experiments. F.L.PARP full length PARP, Cl.PARP cleaved PARP. S.D. is shown in red. Fisher’s exact test was performed on the number of categorical variables scored for panel **A**, unpaired t-test (Welch’s correction) was performed for **B** and **D** and One-way ANOVA (Tukey’s multiple comparison) was performed for **C**. *p < 0.05, **p < 0.01, ***p < 0.001, ****p < 0.0001.

We next speculated that miR-218-5p, by blocking mitophagy and consequently by accumulating damaged mitochondria, may favour the efficacy of DXR treatment. To test this hypothesis, we scored the altered/condensed nuclei in transfected cells with vectors encoded GFP alone or GFP/miR-218-5p, identified with the green signal, and treated with DXR. We found a slight increase in the occurrence of pyknotic nuclei when miR-218-5p was expressed, suggesting an increase in cell death (Fig. 5A). In addition, in cells treated with DXR, we found a slight increase of PARP cleavage (a well-known substrate of caspases) in cells expressing GFP/miR-218-5p, compared to cells expressing GFP alone (Fig. 5B). Consistent with these results, by performing a colony formation assay, we found that a combination of DXR and miR-218-5p reduces the colony formation ability of MCF7 compared to DXR treatment alone (Fig. 5C). Of note, both miR-218-5p or shParkin expression alone did not inhibit MCF7 proliferation in absence of DXR treatment (Supplementary Fig. 3A), nor altered COXII and COXIV levels or PARP cleavage (Supplementary Fig. 3B–E). In a similar manner, the cleavage of PARP was slightly increased in MDA-MB-231 expressing miR218-5p and treated with DXR compared to DXR treatment alone (Fig. 5D).

**Fig. 6:**
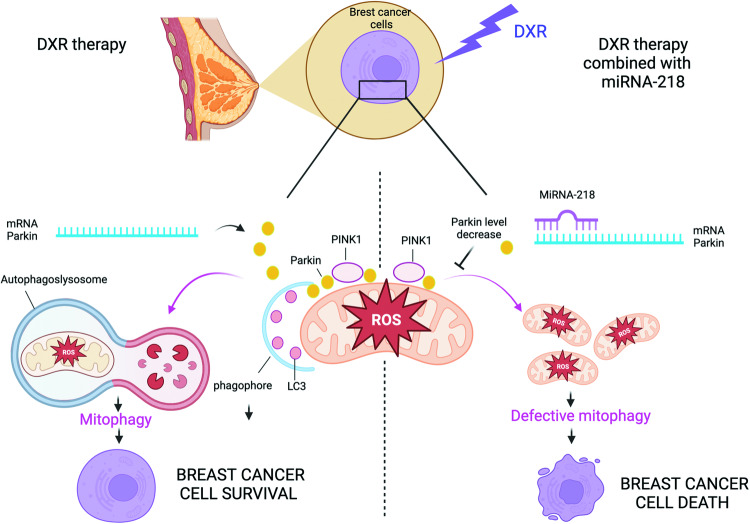
miR-218-5p inhibits DXR-mediated mitophagy and enhances DXR sensitivity of BC cells. Schematic representation of the model proposed in this work. DXR treatment induces mitophagy in breast cancer cells mediated by PINK1 and Parkin. Targeting Parkin by re-introducing miR-218-5p, inhibits DXR-induced mitophagy, thereby enhancing the cytotoxicity of the drug. Created with BioRender.com.

Taken together, our results indicates that a combined administration of miR-218-5p and DXR increased the efficacy of the drug in models of BC characterised by a different aggressiveness (Fig. 6).

## Discussion

To date, DXR represents a recurrent choice for the treatment of BC. In the present study, we investigated whether mitophagy could be a process induced following DXR treatment in BC cells and the possibility to target it through miR-218-5p, in order to improve the efficacy of DXR in BC. We discovered that DXR acts as an inducer of the autophagic mitochondrial clearance mediated by the canonical PINK1/Parkin components in BC cells. Following a microarrays analysis performed in MCF7 cells treated with DXR, we found some deregulated genes involved in mitophagy. Interestingly, we observed the up-regulation of TOMM complex components and the down-regulation of MFN1 and MNF2 encoding for mitofusin1 and 2, both actively degraded after mitophagy induction and required to maintain mitochondrial network. These results suggest that a possible long-term depletion of MFN1 and MFN2 may improve the mitochondria degradation. On the other hand, increased level of TOMM proteins, known to be able to revert PINK1/Parkin-mediated mitophagy [25], could limit the uncontrolled and extensive degradation of mitochondria. Differently, the up-representation of PINK1 mRNA in polysomal fractions showed in the in silico analysis (Supplementary Fig. S1), suggests the idea of an active request of PINK1 protein expression under DXR treatment. Similarly to what was demonstrated for ULK1 mRNA after prolonged autophagy induction [26], we can speculate that a pool of PINK1 mRNA is “ready” on polysome fractions in order to quickly compensate for the PINK1 protein degradation that occurs with damaged mitochondria within lysosomes, during a strong episode of mitophagy.

In accordance with our data, a recent discovery indicates that PINK1-dependent mitophagy is required for inducing and maintaining a drug-tolerant state in lung adenocarcinoma [27]. Moreover, it has been observed that PINK1/Parkin-mediated mitophagy inhibition enhances the efficacy of betulinic acid analogue in multidrug-resistant cancers [28]. Differently, BNIP3-mediated mitophagy is associated with the cisplatin-resistance in osteosarcoma and ovarian cancer [12]. Of note, in our BC cell models, BNIP3 expression results slightly down-regulated (Fig. 1) compared to PINK1 expression. Given that BNIP3L/NIX-dependent mitophagy is induced in colorectal cancer stem cells in response to DXR [10], it is likely that the pathway mediated by PINK1 and Parkin is the one mainly induced by DXR in BC cells. These data support the idea that different mitophagy pathways may be activated in response to drugs/treatments, but also depending on the cellular context.

Despite Parkin being expressed at low level in BC cells [11, 29] and dispensable in lung cancer for chemotherapy-induced mitophagy [11], our silencing experiments of Parkin mRNA indicate its crucial role in DXR-induced mitophagy in BC cells. Our observation of the mitophagy induction after DXR treatment in different models of BC (MCF7, MCF7 mammospheres and MDA-MB-231), suggests that activation of the mitophagy process following DXR treatment is a conserved mechanism in BC cells. Since miR-218-5p re-expression is capable of blocking DXR-induced mitophagy in our models of BC, our results highlight Parkin as an interesting target in BC cells in response to DXR treatment. Moreover miR-218-5p, originally characterised as regulator of motoneurons development [30–32] and found to be reduced in tumour tissues compared to the adjacent normal ones [16, 17], it has been proposed as a low-risk factor of BC [33, 34]. Moreover, miR-218-5p reintroduction is sufficient per se to limit migration and proliferation of MCF7[16] and of MDA-MB-231 cells [17]. Although we did not find any anti-proliferative effect of miR-218-5p per se, as described previously [16], we observed a synergic effect of DXR and miR-218-5p. These findings are in agreement with Hu and colleagues who described a cytotoxic effect of miR-218-5p with DXR or Paclitaxel in the context of BC [35]. Interestingly, it has been demonstrated that Paclitaxel reduces PINK1-mediated mitophagy in TBNC cell lines [36], highlighting that inhibition of mitophagy favours the chemotherapy effect. These evidence supports the intriguing idea that the combined therapy Paclitaxel/DXR may recapitulate the co-treatment with miR-218-5p. Our findings account for a future direction in studying the effects of the co-administration of Paclitaxel and miR-218-5p on the mitochondria quality control and in characterising the main cell death pathway activated in treatments combining miR-218-5p and chemotherapy.

In conclusion, our results showed the involvement of PINK1/Parkin mitophagy in the response of BC cells to DXR therapy, suggesting a mitochondrial quality control as a new possible target. Considering the current challenges associated to refractory treatment for BC, our work could provide a possible approach to improve the efficacy of chemotherapy, to prevent tumour resistance. In addition, because both miRNA and DXR are efficiently delivered by nano-carriers [37], our study could potentially lead to the development of an innovative nano-technological approach for combination therapy.

## Material and methods

### Cell cultures and treatments

The MCF7 luminal A phenotype and MDA-MB-231 triple negative breast cancer cells (kind gift of dr. Venturina Stagni, IBPM-CNR) were grown at 37 °C and 5% CO2 in complete DMEM (Dulbecco’s Modified Eagle Medium, GIBCO/Thermo Fisher Scientific, Waltham, Massachusetts, USA, 41966-029) high glucose supplemented with 10% foetal bovine serum (FBS), and routinely tested for mycoplasma contamination. For DXR (Selleck Chemicals, Houston,Texas, USA, S1208) administration, cells were treated as follows: (a) at 30 μM for 24 h in order to analyse mitophagic gene expressions and to evaluate mitophagy induction; at 30 μM for 1 or 2 h in order to analyse PINK1 stabilisation and GFP-Parkin translocation on mitochondria, respectively; (b) at 10 μM for 48 h in order to count the number of pyknotic nuclei; (c) at 2 μM for 72 h to analyse mitophagy in mammospheres; (d) at 1 μM for 12 h to impair colony formation abilities of MCF7 cells and to visualised cleaving of PARP. To analyse mitochondrial ROS production by flow cytometry, cells were incubated with MitoSOX Red probe (Invitrogen/Thermo Fisher Scientific, M36008) for 15 min at 37 °C. Doxycycline (Clontech, Mountain View, California, USA, 631311) at 2 µg/mL was added to transfected cells in order to induce miR-218-5p expression 24 h before mitophagy induction. Autophagosome–lysosome fusion was blocked with NH_4_Cl (Sigma-Aldrich/MERCK, Darmstadt, Germany, 09718) at 20 mM for 24 h.

### Plasmids and transient transfections

The Tet-O-FW GFP/miR-218-5p (indicated as miR-218-5p in the graphs and the pictures of the different figures) construct generation is described in ref. [19] and was transfected in combination with the plasmid encoded for the rtTA transactivator. An empty Tet-O-FW GFP vector (kind gift of dr. GianCarlo Bellenchi, IGB-CNR) was used as control. Transient transfections in MCF7 and MDA-MB-231 cells were performed using polyethylenimine (PEI, Tebu-Bio, Le Perray-en-Yvelines, France, 23966-1). Other plasmids used in this study are vectors encoded for GFP-Parkin and ShortHairpin(sh)-Parkin/GFP. A vector encoding for GFP alone was used as a control.

### Mammosphere cultures

MCF7 and derivate cell lines were grown in ultralow attachment 6-well plates (Corning, Glendale, Arizona, USA, 3471) at a density of 4000 cells/mL in mammosphere medium [Dulbecco’s modified Eagle’s medium/F- 12, containing 5 μg/mL insulin (Sigma-Aldrich, I6634), B27 (Invitrogen, 12587010), 20 ng/ml epidermal growth factor (Sigma-Aldrich, 324331), 10 ng/ml basic fibroblast growth factor (ProteinTech, Cranbury, New Jersey, USA, 100-18B) and 0,4% Bovine Serum Albumine (Sigma-Aldrich)] as described in ref. [38]. After at least 6-7 days, the mammospheres were treated with DXR for the indicated times and concentrations (see “Cell cultures and treatments”). Mammosphere pellets were collected by gentle centrifugation (900 rpm, 5 min) for total RNA and DNA extraction. Images were captured with Zoe Fluorescence Cell Imager (Biorad, Hercules, California, USA).

### Western blotting

Cells were lysed in RIPA buffer (150 mM NaCl, 50 mM Tris with pH 7.4, 1% Triton, 0.5% Nonidet P40, 10% glycerol, and 2.5% sodium deoxycholate) supplemented with protease inhibitors (Roche Diagnostics, Basel, Switzerland, 11836153001). Proteins were resolved by SDS PAGE and transferred on a PVDF membranes (Millipore/MERCK, Darmstadt, Germany, Immobilon-P IPVH00010). About 20–30 μg of extract per lane were loaded. Blocking and HRP-conjugated secondary antibodies (Biorad) incubations were performed at room temperature in TBS containing 0.1% Tween and 5% low fat milk or 1 h. Fluorescent signals were revealed using chemo-luminescent HRP substrate (Millipore, Immobilon, WBKLS0500). Primary antibody incubations were carried out overnight at +4 °C. Antibodies used were: rabbit anti-β actin (Sigma Aldrich, A2066), mouse anti-COXII (Abcam, Cambridge, United Kingdom, ab110258), rabbit anti-PINK1 (Novus Biologicals, Centennial, Colorado, USA, BC100-494), mouse anti-GAPDH (Sigma Aldrich, SAB1405848), rabbit anti-Parkin (Cell Signalling Technologies, Danvers, Massachusetts, USA, 2132), mouse anti-COXIV (Abcam, ab33985), mouse anti-vinculin (Santa Cruz Biotechnology, Dallas, Texas, USA, 7F9, sc-73614), mouse anti-TOMM20 (Santa Cruz Biotechnology, sc-17764), rabbit anti-PARP (Cell Signalling Technologies, 9542).

### Mitochondrial fractioning

To analyse GFP-Parkin translocation on mitochondria, subcellular fractioning has been performed at 4 °C according to [39], with some modifications. Briefly, MCF7 cells were re-suspended in a buffer containing 210 mM mannitol, 70 mM sucrose, 5 mM tris-HCl pH 7.5 and 1 mM EDTA pH 7.5, plus protease inhibitors (Roche Diagnostics, 11836153001). Cell pellet was passed through a 26-gauge needle for 30 strokes, to disrupt cell membrane integrity. The resulting extract was firstly centrifuged at 1500 × *g* for 5 min, to remove nuclei and cellular debris, and then a second centrifugation was carried out at 10,000 × *g* for 15 min to pellet mitochondrial fraction. The supernatant contained the cytoplasmic fraction.

### Immunofluorescence

Cells grown on coverslips were fixed with 3.7% formaldehyde in PBS, 10 min at room temperature, followed by permeabilisation in PBS containing 0.1% TritonX-100, 5 min at room temperature. Blocking and incubations with secondary antibodies were performed at room temperature in PBS containing 0.05% Tween and 3% BSA. Primary antibodies used were: mouse anti-TOMM20 (Santa Cruz Biotechnology, sc-17764), rabbit anti-LC3A/B (Cell Signalling Technologies, 12741S). Cells were counterstained with 4,6-diamidino-2-phenylindole (DAPI, 0.1 μg/ml; Sigma-Aldrich) and mounted using Flouromount Mounting Media (Sigma-Aldrich, F4680). Samples were analysed using a Zeiss LSM 800 microscope equipped with 63X or 100X (oil immersion) objectives. Images were acquired using ZEN system (ZEISS, Germany). ImageJ software was used for image analysis. Calculation of the mitochondrial content as percentage of cell area occupied by mitochondria, was performed using the “Mitophagy” macro [40].

### Colony forming assays

Transfected MCF7 cells were treated with DXR at the indicated doses and timings (see “Cell cultures and treatment”), collected, counted and then plated at the density of 5000 cells/Petri dishes. Cells were grown for fourteen days in the absence of drug. Surviving colonies were fixed and stained with Cresyl Violet (Sigma-Aldrich, C0775) 0.5% in methanol 20%, air-dried, scanned and counted with ImageJ software using the plug-in “Analyse particles”.

### Real-time PCR

Gene expression analysis was performed on total RNA, isolated from cells using the ReliaPrep™ RNA Cell Miniprep System (Promega, Madison, Wisconsin, USA, Z6011) or RNeasy Mini Kit (QIAGEN, Hilden, Germany, QG74104), according to the manufacturer’s instruction. 1 μg of RNA has been retro-transcribed in cDNA using GoScript Reverse Trascription Mix, Random Primers (Promega, A2801). To quantified the relative mtDNA content in mammospheres, the total DNA from mammospheres has been extracted using Quick-DNA MiniPrep (ZymoResearch, Irvine, California, USA, D3024) or QIAamp DNA Micro Kit (QIAGEN, 56304). PCR reactions were performed with Power Up SYBR Green Master Mix (Applied Biosystems/Thermo Fisher Scientific, Waltham, Massachusetts, USA, A25741) on a LightCycler 480 thermo-cycler (Roche). mRNA level and mtDNA/nDNA ratio were calculated with the ΔΔCt method, after normalisation with β-2-microglobulin (B2M) mRNA for gene expression and GAPDH gene for mtDNA levels, used as housekeeping. The Ct of c/n DNA samples for each experiment was the mean of at least two technical replicate values of Ct for each sample.

**Table:** 

Gene	Forward	Reverse
PRKN	5’-GGGTCGTGAACAAACTGCCGATCATT-3’	5’-AGGAGCCCCGTCCTGGTTTT-3’
B2M	5’-CTCCGTGGCCTTAGCTGTG-3’	5’-TCTCTGCTGGATGACGTGAG-3’
MT-COXII	5’-GTCCTGTATGCCCTTTTCCTAACACTC-3’	5’-GACCTCGTCTGTTATGTAAAGGATGCG-3’
GAPDH [41]	5’-TTCAACAGCGACACCCACTC-3’	5’-CGCCAGACCCTGCACTTTTT-3’

### Mitochondrial superoxide measurements by flow cytometry

Mitochondrial superoxide was measured using a previously described flow cytometry method [42]. Briefly, after the different treatments, cells were washed in PBS and stained with and MitoSOX Red (Thermo Fisher, M36008) for 30 min at 37 °C in the dark and at a final concentration of 5 μM according to manufacturer’s instructions. Then, cells were washed with PBS and stained with Live dead NIR dye (Thermo Fisher, L34975) for 10 min in the dark to assess cell viability. Finally, cells were washed twice with PBS and acquired at CytoFLEX flow cytometer (Beckman Coulter) following gating out cell debris, doublets and dead cells with FlowJo software (Treestar, Ashland, OR, USA).

### In silico analysis of other datasets

We selected for analysis relevant datasets from the ArrayExpress (https://www.ebi.ac.uk/biostudies/arrayexpress) and Gene Expression Omnibus (https://www.ncbi.nlm.nih.gov/geo/) repositories. All the datasets, concern MCF7 cells, except E-GEOD-37543 that contains doxorubicin-treated human cancer tissues: E-GEOD-19638, E-GEOD-23399, E-GEOD_26599, E-GEOD-24065, E-GEOD_33055, E-GEOD-36870, E-GEOD-27254, E-GEOD_50650, E-MTAB_1643, E-GEOD-37543. Most of these datasets allow to compare doxorubicin-treated MCF7 cells vs untreated MCF7 cells and in one case (E-GEOD-27254) to compare Doxorubicin-resistant MCF7 cells to DXR-sensitive MCF7 cells. Filtered normalised expression matrices were downloaded and the differentially expressed genes (DEGs) doxorubicin -treated MCF7 cells vs control cells were selected by the limma package in R-Bioconductor, using the same Log2 fold-change ratio (>1.0) and FDR (<0.05) thresholds. The selected DEG lists were then matched against 4 reference mitophagy-related and 2 doxorubicin-related gene lists extracted from databases using keywords: Gene Ontology hsa: “autophagy of mitochondrion” (46 genes) and “mitophagy” (9 genes); Reactome pathways: “mitophagy” (29 genes); KEGG pathways hsa: “mitophagy” (68 genes); GeneCards: “Doxorubucin pathway” (28 genes); PharmGKB: “Doxorubucin pathway” (18 genes).

### Microarray gene expression profiling

The purity of total RNA, isolated from the MCF7 using the RNeasy Mini Kit, and integrity were determined using the Agilent 2100 Bioanalyzer (Agilent Technologies, Santa Clara, California, USA). Samples were loaded onto the Eukaryote total RNA 6000 nano Kit (Agilent Technologies,). The RNA integrity number (RIN) for all the samples was calculated and samples with a value lower than 8.0 were discarded. The gene expression profiling was performed using the standard protocol for Agilent one-colour gene expression microarray (One-Colour Microarray-Based Gene Expression Analysis ver 6.9), using Agilent SurePrint G3 Human GE v3 8x60K chip (Grid ID 072363).

### Microarray data analysis

Data extraction from the Agilent scanner images was accomplished by Feature Extraction software version 12.0. Data filtering, normalisation and analysis were performed using R-Bioconductor (data are available from Gene Expression Omnibus with entry GSE244574). All features with the flag gIsWellAboveBG = 0 (too close to background) in at least one sample have been filtered out, data were normalised to the 75th percentile. DEGs were selected using R-Bioconductor by a combination of Log2 fold-change ratio (>1.0) and FDR (<0.05) thresholds by the limma package. Pathway analysis of gene lists was performed using Gene Set Enrichment Analysis (GSEA, https://www.gsea-msigdb.org/gsea/index.jsp), other online tools and by the Fisher’s exact test in R. Accession ID: GSE244574.

### Statistical analysis

Data from at least three independent biological replicates (represented by solid black dots in the histograms) are expressed as mean ± standard deviation (S.D.), depicted in red in the histograms. The sample size, indicated in figure legends, was not pre-determined. No blinded analysis were performed. All statistical tests, indicated in the respective figure legends, were performed with GraphPad Prism 8 software (San Diego, California, USA). The unpaired t-test and the ordinary one-way ANOVA multiple comparison test or the Mann-Whitney test and the Kruskal-Wallis test were used for measurements of continuous variables, depending on whether the samples were normally or non-normally distributed (assessed by the Shapiro-Wilk normality test); Fisher’s exact test was used for analyses of categorical variables. Statistical significance (*) was set at *p* < 0.05.

### Supplementary information

Suppl Figure 1

Suppl Figure 2

Suppl Figure 3

Suppl Figure legends

Uncropped Western-blots

## Data Availability

Microarray data are available from Gene Expression Omnibus with the entry GSE244574. Uncropped original western blots used in this manuscript can be found in ‘Supplemental Material’. All data generated during this study and supporting the present results are available from the corresponding author upon reasonable request.
