# Supplementary figures and images for: miR-218-5p and doxorubicin combination enhances anticancer activity in breast cancer cells through Parkin-dependent mitophagy inhibition
Source: Cell Death Discov. 2024 Mar 21;10:149. doi: 10.1038/s41420-024-01914-7 (PMC10957887; doi:10.1038/s41420-024-01914-7)

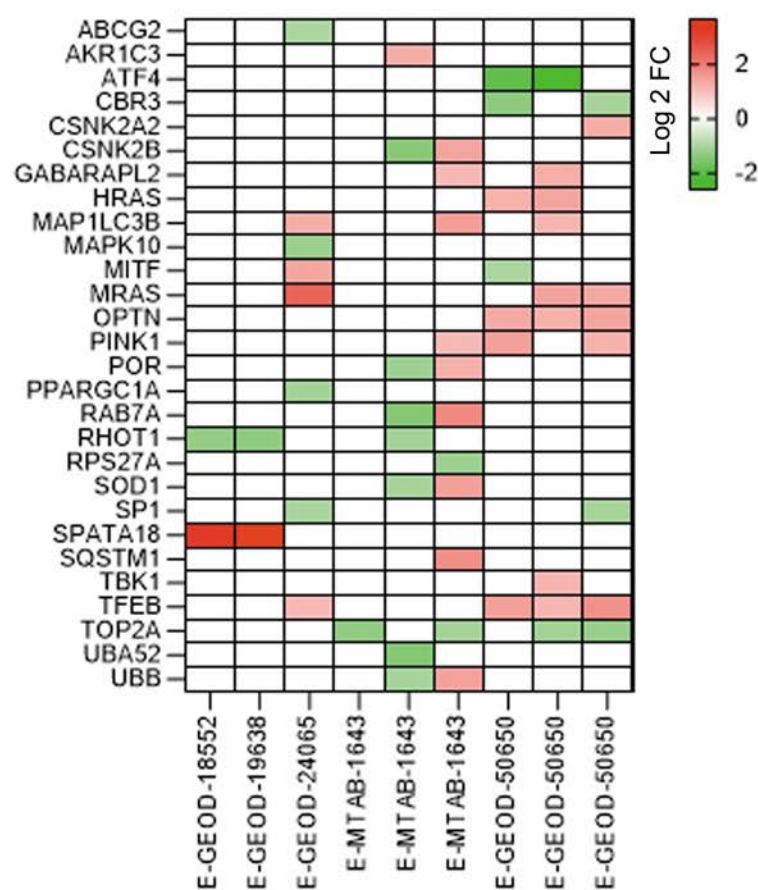

Supplementary figure 1

Supplement: Supplementary file 1 — Suppl Figure 1 [file 41420_2024_1914_MOESM1_ESM.pdf]

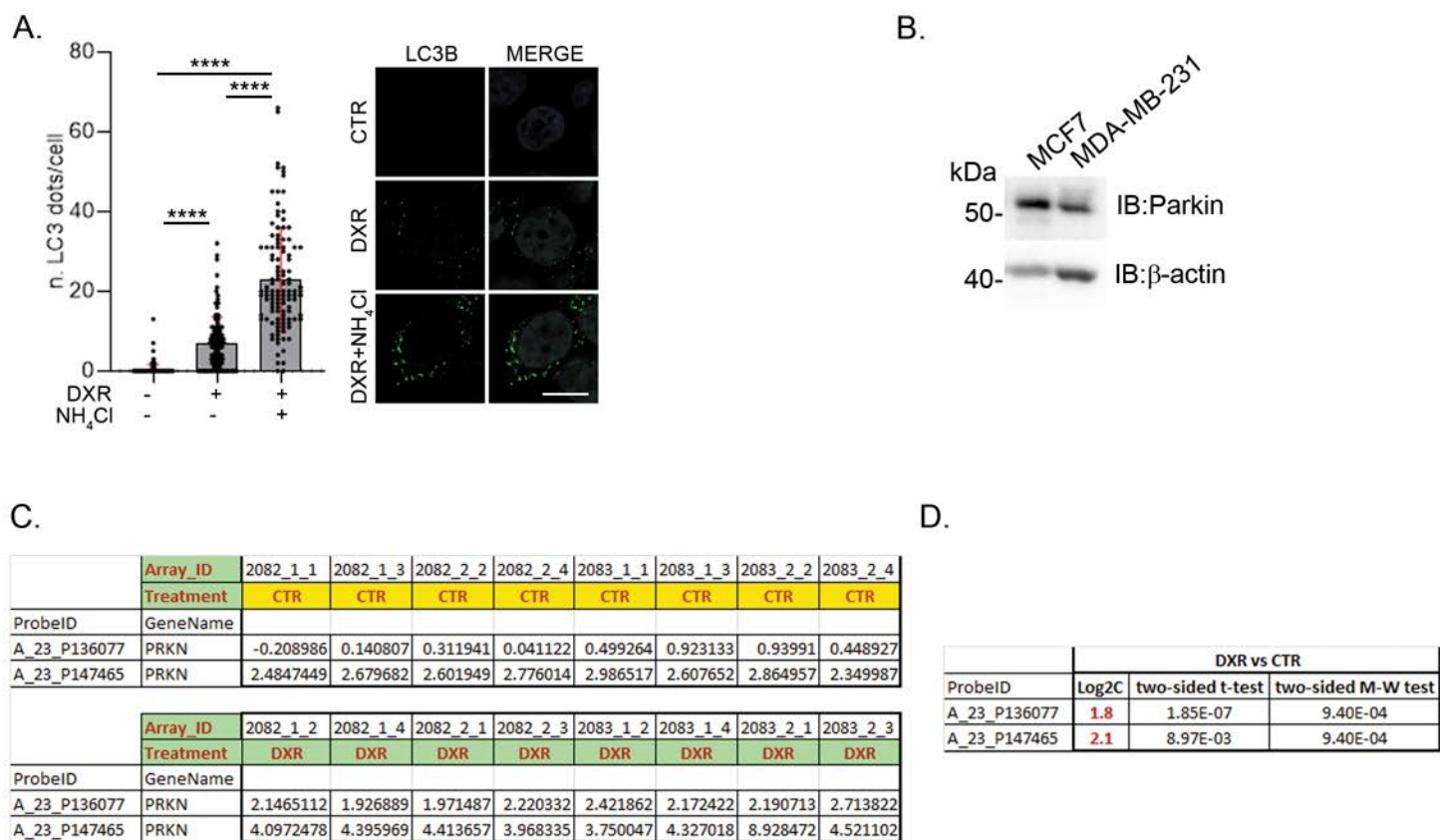

Supplementary figure 2

Supplement: Supplementary file 2 — Suppl Figure 2 [file 41420_2024_1914_MOESM2_ESM.pdf]

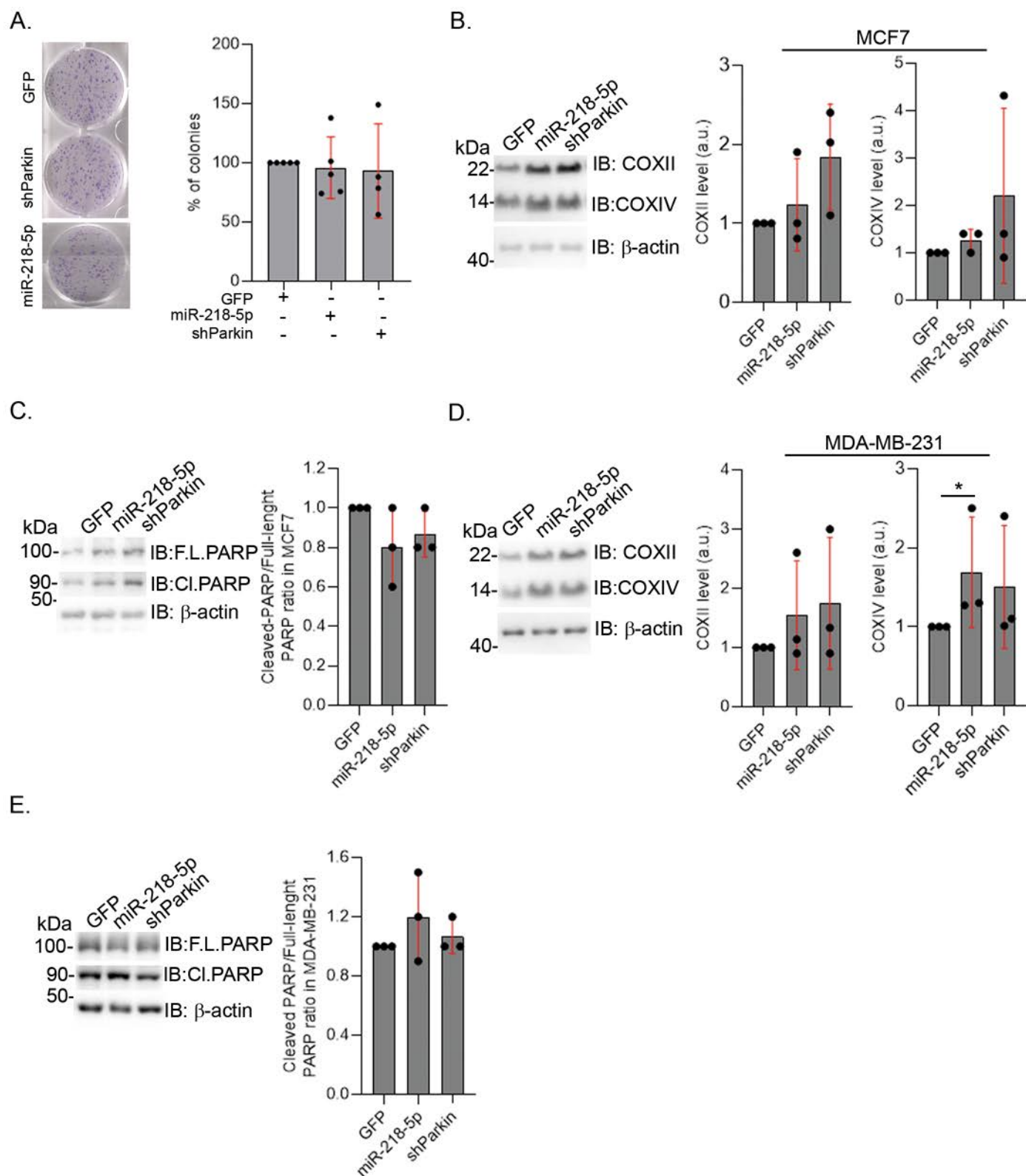

**Supplementary figure 3**

Supplement: Supplementary file 3 — Suppl Figure 3 [file 41420_2024_1914_MOESM3_ESM.pdf]

2C

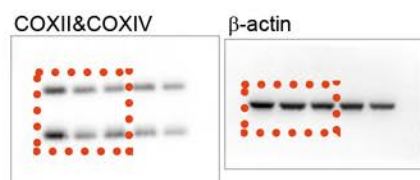

2D

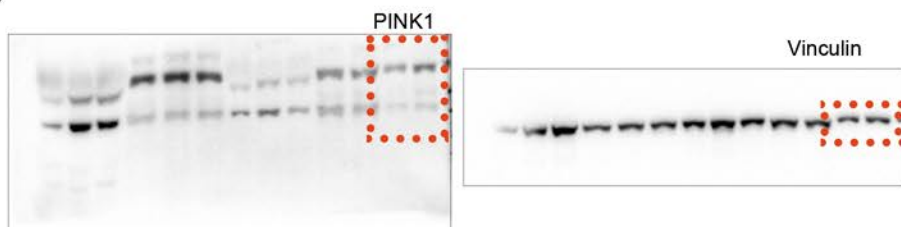

2E

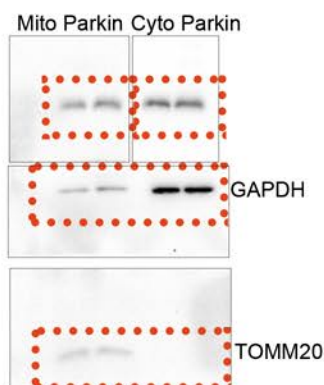

3B

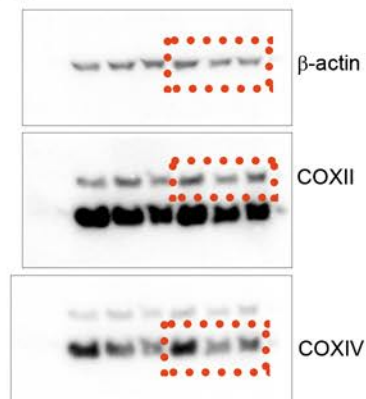

4A

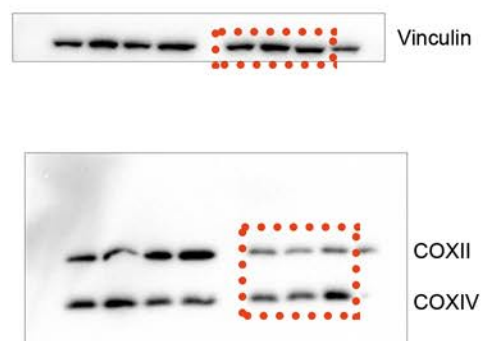

4D

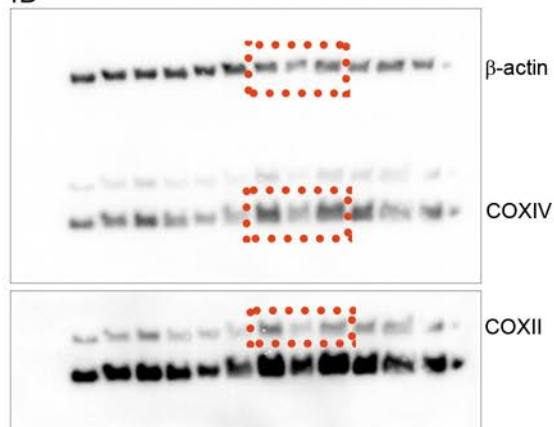

5B

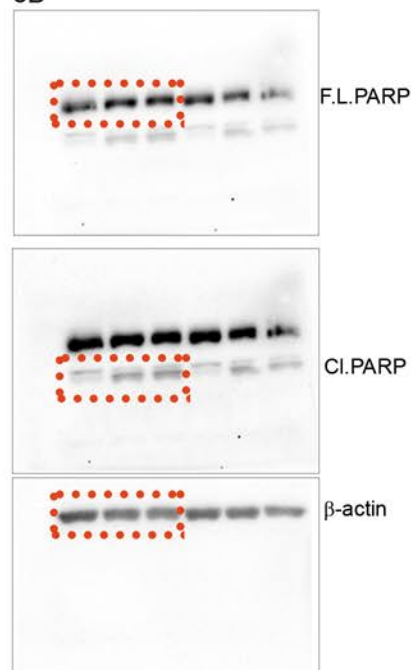

5D

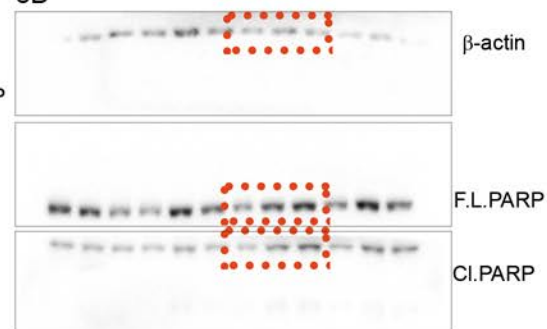

S2B

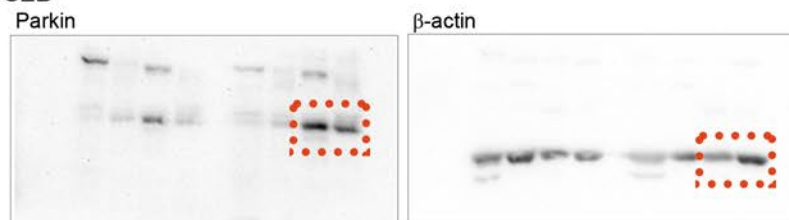

S3B,D

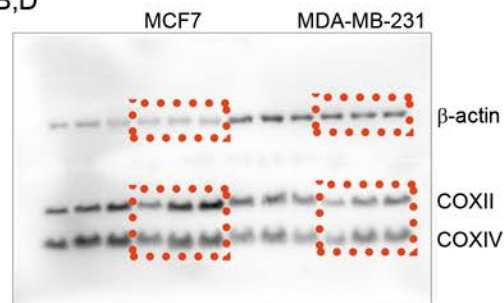

S3C,E

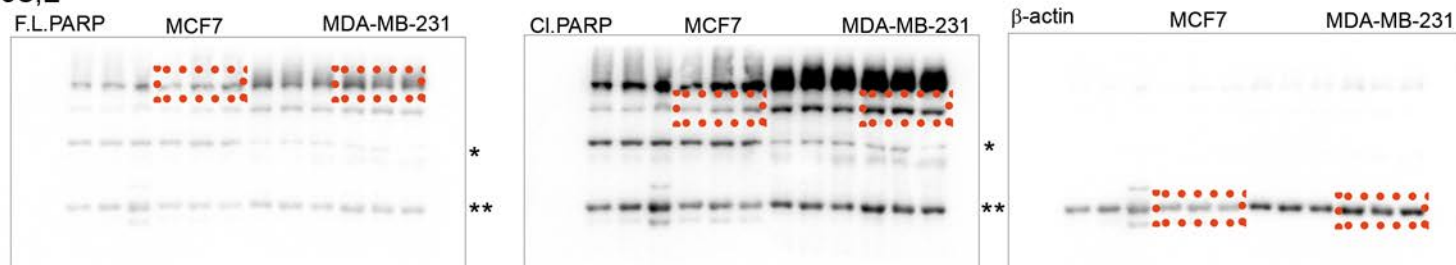

\*=additional stain

\*\*=β-actin stain

Supplementary uncropped WB

Supplement: Supplementary file 5 — Uncropped Western-blots [file 41420_2024_1914_MOESM5_ESM.pdf]
