# Supplementary material for: miR-218-5p and doxorubicin combination enhances anticancer activity in breast cancer cells through Parkin-dependent mitophagy inhibition
Source: Cell Death Discov. 2024 Mar 21;10:149. doi: 10.1038/s41420-024-01914-7 (PMC10957887; doi:10.1038/s41420-024-01914-7)
Supplement: Supplementary file 4 — Suppl Figure legends [file 41420_2024_1914_MOESM4_ESM.docx]

**Supplementary Figure Legend**

**Supplementary Figure 1.** Heatmap shown down (green) and up (red) regulated genes expressed (FDR < 0.05; Log2 fold-change ratio>1.0) after DXR treatment from published datasets (listed below the heatmap), filtered for selected keywords (for details, see Materials and Methods). For both heatmaps white squares are genes not listed in the referred gene list.

**Supplementary Figure 2.** A. The induction of the autophagosomes formation by DXR treatment 30 µM for 24 hours is quantified using an antibody against LC3 protein. The accumulation of LC3 dots following NH_4_Cl administration is evidenced. Each dot represents the real value of the number of LC3 dots inside each cell analysed. At least 116 cells were analysed, from 3 independent experiments. Representative images of the different conditions are illustrated on the right panels. Scale bar, 10 µM is shown. Statistical analysis was performed using the Kruskal-Wallis test (Dunn’s multiple comparison) ****: p < 0.0001. B. Representative images of western blot showing Parkin level in both MCF7 and MDA-MB-231 BC cell lines. C. Parkin gene expression level in Log2 scale, in unfiltered data, for doxorubicin-treated (DXR) and untreated (CTR) samples.

**Supplementary Figure 3** A. Colony formation assay highlights the ability of cells to form colonies in cells expressing GFP, GFP/shParkin or GFP/miR-218-5p. The graph represents the % of colonies respect to the control condition. Solid dots represent the value of the indicated conditions, from at least 4 independent experiments. S.D. is shown in red. B-E. Total lysate from MCF7 and MDA-MB-231 cells transfected with GFP, GFP/miR-218-5p or GFP/shParkin vectors were immunoblotted with the indicated antibodies. The graphs show the levels of mitochondrial markers COXII and COXIV, and cleaved PARP, normalized on β-actin. Solid dots represent the value respect to the control condition, from 3 independent experiments. F.L.PARP: full length PARP; Cl.PARP: cleaved PARP. S.D. is shown in red. Statistical analysis was performed using one-way ANOVA (Tukey’s multiple comparison); Kruskal-Wallis test (Dunn’s multiple comparison) for COXIV levels; *: p < 0.05.
